# Clinical advantages of incorporating predicted weekly anatomy in IMPT optimization with reduced setup error

**DOI:** 10.1002/mp.17412

**Published:** 2024-09-19

**Authors:** Ying Zhang, Mark Ka Heng Chan

**Affiliations:** ^1^ Department of Medical Physics and Biomedical Engineering University College London London UK; ^2^ Department of Radiation Oncology University Nebraska Medical Center Omaha USA

**Keywords:** application of anatomical modeling, head and neck cancer, intensity‐modulated proton therapy, robust optimization, uncertainty management

## Abstract

**Background:**

In head and neck (H&N) cancer treatment, a conventional setup error (SE) of 3mm is often used in robust optimization (cRO3mm). However, cRO3mm may lead to excessive radiation doses to organs at risk (OARs) and does not purposefully compensate for interfractional anatomy variations.

**Purpose:**

This study introduces a method using predicted images from an anatomical model and a reduced 1mm SE uncertainty for robust optimization (aRO1mm), aiming to decrease the dose to OARs without affecting the coverage of the clinical target volume (CTV).

**Methods:**

This retrospective study involved 10 nasopharynx radiotherapy patients. Validation CT scans (vCT) from treatment weeks 1 to 6 were analyzed. A predictive anatomical model, designed to capture the average anatomical changes over time, provided predicted CT images for weeks 1, 3, and 5. We compared three optimization scenarios: (1) aRO1mm, using three predicted images with 1mm setup shift and 3% range uncertainty, (2) cRO3mm, with a robust 3mm setup shift and 3% range uncertainty, and (3) cRO1mm, a robust 1mm setup shift and 3% range uncertainty. The accumulated dose to CTVs and serial organs was evaluated under these uncertainties, while parallel OARs were assessed using the accumulated nominal dose (without errors).

**Results:**

The accumulated volume receiving 94% of the prescribed dose (V94) for CTVs in cRO3mm exceeded 98%, meeting the clinical goal. For high‐risk CTV, the minimum V94 was 96.44% in aRO1mm and 94.05% in cRO1mm. For low‐risk CTV, these values were 97.68% in aRO1mm and 97.15% in cRO1mm. When comparing aRO1mm to cRO3mm on OARs, aRO1mm reduced normal tissue complication probability (NTCP) for grade ≥2 xerostomia and dysphagia by averages of 3.67% and 1.54%, respectively.

**Conclusion:**

aRO1mm lowers the radiation dose to OARs compared to the traditional approach, while maintaining adequate dose coverage on the target area. This method offers an improved strategy for managing uncertainties in radiation therapy planning for H&N cancer, enhancing treatment effectiveness.

## 1 INTRODUCTION

In the treatment of head and neck (H&N) cancer, the proximity of cancer cells to critical organs and structures such as the parotid glands, oral cavity, brainstem, and pharyngeal constrictor muscles presents a challenge to radiotherapy. Radiation dose in these areas can result in serious damage to these sensitive structures, leading to complications like dysphagia (swallowing difficulty) and xerostomia (dry mouth) that compromise patients' quality of life over the long term. Intensity‐modulated proton therapy (IMPT) as the state‐of‐the‐art radiotherapy technique has advantages in delivering a conformal dose to the target while minimizing the dose to the adjacent normal tissue,^1^, ^2^, ^3^ exploiting the steep falloff of the Bragg peak. The potential benefits of proton therapy over photon therapy in H&N cancer treatment have been demonstrated in a few dosimetric and clinical studies.^2^, ^3^ However, this precise delivery technique has inherent sensitivity to various uncertainties prevalent in H&N cancer treatment.

The uncertainty primarily comes from three aspects: (1) Inaccuracies in CT imaging, such as noise, artifacts, beam hardening, and density heterogeneity, lead to errors in Hounsfield Units (HU), which will be converted to relative stopping power (RSP) for dose calculation. These inaccuracies, referred to as range uncertainties,^4^, ^5^, ^6^ are typically modeled by uniformly altering HU values by a few per cent; (2) the variations from beam reproducibility and patient positioning. Setup uncertainty is generally modeled by a few millimetres of rigid shift; (3) anatomical changes during the treatment, including small nonrigid variations (sNRVs) and progressive changes. sNRVs refer to motions that have no trends to follow,^7^, ^8^ such as nasal filling, jaw movement, neck folds, spine flexion, and shoulder position changes. Progressive changes include tumor progression/shrinkage, gland shrinkage, weight loss, and so forth.^9^, ^10^, ^11^, ^12^ Yan et al.^11^ showed weight loss, which is often accompanied by the shrinkage of the patient's outline, at 25 fractions is 3.9%–25.5% due to complications such as dysphagia (swallowing difficulties) and dysgeusia (taste changes). What follows are changes in the positions of the tumor and OARs. Bhide et al.^12^ showed the parotid volume of 20 H&N patients decreased with a reduction rate between 21.3% and 42%, and an average of 2.3 mm medial shift occurred by the fourth week of treatment. There are models of these anatomical changes.^7^, ^10^, ^13^, ^14^, ^15^, ^16^, ^17^, ^18^

Image‐guided radiotherapy (IGRT) uses the online image‐guided six‐degree correction to control the position uncertainty. A few studies and institutions use 1mm as the position uncertainty.^5^, ^19^, ^20^, ^21^ In conventional optimization, the process includes setup and range uncertainties of 3mm and 3% for H&N cancer patients.^22^, ^23^ This optimization strategy, referred to as cRO3mm, is used as an empirical measure to account for the interfraction anatomical variability.^20^, ^21^ However, the anatomical variations that occur during treatment are not uniform across all directions, which means that applying a uniform 3mm shift might inadvertently result in excessive radiation dose to the organs at risk (OARs).

When the magnitude of setup errors in robust optimization is reduced, plans would likely be more sensitive to anatomical changes. The average model (AM) proposed by Zhang et al.^10^ can capture the systematic interfractional changes based on population data, offering a way to account for systematic anatomical uncertainty. In this study, we simulated the positioning shift of patients of 1mm during the treatment. We aim to: (1) Show that using the actual position error of 1mm in robust optimization, referred to as cRO1mm, cannot help with tumor coverage degradation caused by anatomical changes during treatment. (2) Demonstrate that the strategy of cRO3mm can guarantee tumor coverage but at the cost of overdose to OARs. (3) Propose using predicted images and the 1mm position error together to explicitly account for geometric uncertainty in robust optimization, a strategy referred to as aRO1mm. Unlike the cRO3mm approach, this approach aims to more accurately reflect the interfraction anatomical variability by predictive modeling, which anticipates changes in patient anatomy over time, thereby improving the robustness of the treatment plans while minimizing unnecessary radiation exposure to OARs. The paper validated the potential and the benefits of incorporating predicted weekly anatomies in IMPT optimization to ensure tumor coverage and reduce the dose of OARs.

## 2 MATERIALS AND METHODS

### 2.1 Average model

We used a leave‐one‐out cross‐validation strategy to build models. One patient was randomly selected from the cohort to serve as the test subject, while the remaining 19 patients constituted the training population. Using this training set, we computed the average weekly deformation model.^10^ This AM was then applied to the excluded patient's planning CT to predict its anatomical changes during treatment. This process was iterated for 10 randomly selected patients, with each iteration maintaining a strict separation between the test subject and the training cohort. Consequently, each validation patient remained independent of the training population used to generate the average model, ensuring an unbiased assessment of the model's generalizability. The patient who experienced the largest weight loss of 18%, and another who had the highest high‐risk CTV reduction of 61% were among the selected patients.

The predictive ability of the model has been validated on CT numbers, contours, proton spot location deviation and dose distribution.^10^ Compared with no model, in which predicted images were replaced by planning CT, the average mean surface distance (MSD) between the predicted parotid contours and the corresponding parotid contours in validation CT (vCT) of parotid glands was reduced from 1.78 mm (no model) to 1.12 mm in the average model at week 5. The average gamma index (using 2 mm/2%) between the dose calculated on predicted CT and real CT at week 5 was improved from 94.1% with 95% confidence interval (CI) of 2.45% for no model to 95.1% with 95% CI of 2.07% for the average model.

### 2.2 Patient data

Contoured CT imaging data of ten advanced nasopharyngeal carcinoma (NPC) patients with primary and bilateral neck target volumes, randomly selected from the previous study of the average model,^10^ were included in this study. All patients had thermoplastic masks covering the head, neck, and shoulders for immobilization in the supine position during the original course of radiotherapy. Their clinical target volumes (CTVs) of the primary tumor, lymph nodes, and elective regions decreased by an average of 1.5%, 2.3%, and 0.3% per treatment day, respectively. The mean three‐dimensional displacements of the CTVs ranged from 2.5 to 3.7 mm. Significant volume reductions were observed in the salivary and thyroid glands: 28.6% ± 14.6% (mean ± standard deviation) for the parotid gland, 26.6% ± 17.1% for the submandibular gland, and 12.3% ± 11.3% for the thyroid gland. The center of mass of the parotid gland shifted medially by 2.0–2.6 mm, while the submandibular gland and thyroid exhibited a less pronounced medial shift of 0.1–0.5 mm. More details of their diseases, original treatments, and evaluation of anatomical changes can be found in ref. [9, 24, 25, 26]. It should be noted that this is a retrospective study using the patients' imaging data and contours. Each patient had planning CT (pCT) and vCT from week 1 to 6 (at fractions of 5, 10, 15,…, 30). The AM,^10^ which was built at each time point to capture progressive changes, was used to generate predicted images of weeks 1, 3, and 5 for each patient. The model is most effective in predicting the patient outline changes. Hence, in aRO1mm, the contours on the predicted images for robust optimization are created by following theses rules: For all OAR contours and the low‐risk CTV (nodal area) affected by neck changes, we use the predicted contours from contour propagation. For the high‐risk CTV, we use the initial CTV of the planning CT in the predicted plan to ensure target coverage.

Three proton plans with different optimization scenarios are implemented in this study: (1) cRO3mm using one pCT with a 3mm setup shift and 3% range uncertainty, (2) cRO1mm using the pCT with a 1mm setup shift and 3% range uncertainty, and (3) aRO1mm with 1mm setup shift and 3% range uncertainty using three predictive images at week 1, 3, and 5. Similar to cRO3mm, aRO extends the uncertainty scenarios to 3 predicted CTs plus pCT. The minimax algorithm seeks a solution that best achieves robust objectives across all scenarios, including different anatomies, setup errors, and range errors. Predicted CT contours are used in the robust function. Detailed explanations can be found in Appendix A.

The dosimetric goals for all plans in this study are summarized in Table 1 for CTVs and serial organs.^27^ For other parallel OARs, our goal is to minimize their dose as much as possible. A plan was deemed acceptable if the clinical goals set for the CTV and serial organs were met in the nominal scenario (the error‐free distribution) and the corresponding uncertainty scenarios for each strategy. The voxmin dose distribution, which records the minimum dose of each voxel under uncertainty scenarios, and voxmax dose distribution, which records the maximum dose of each voxel under uncertainty scenarios, are used for dose metric evaluation. Replanning will be triggered when CTV V94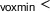 94%.

**TABLE 1:** Dosimetric goals of the treatment plans created in this study.

Structure	Clinical goal under 3mm/3% uncertainty	
high‐risk CTV (Prescription: 70Gy, 35 fractions)	V94 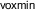 (The percentage of CTV volume received at least 94% of prescription dose in voxmin dose distribution) > 98%	1
low‐risk‐CTV (Prescription: 54.25Gy, 35 fractions)	V94 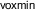 > 98%	1
CTV	D2 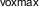 (The voxmax dose to the hottest 2% volume) < 110% of prescription dose (77Gy)	1
Brainstem	${D0.03\mathrm{cc}}_{\text{voxmax}}$ (The voxmax dose to the hottest 0.03 cubic centimetre) <68 Gy	2
Spinal cord	${D0.03\mathrm{cc}}_{\text{voxmax}}$ <58.5 Gy	2
Optic nerves and chiasm	${D0.03\mathrm{cc}}_{\text{voxmax}}$ <64 Gy	2
Structure	Clinical goal without uncertainty	Priority
Brainstem	D0.03cc (The nominal dose to the hottest 0.03 cubic centimetre)<63.1 Gy	2
Spinal cord	D0.03cc <55 Gy	2
Optic nerves and chiasm	D0.03cc <59.5 Gy	2

Abbreviation: CTV, clinical target volume.

All aRO and cRO plans were generated in a research version of RayStation (v.11B, RaySearch, Stockholm, Sweden) for the IBA Proteus Plus machine, leveraging pencil beam spot scanning technology. A standard six‐beam arrangement for NPC was adopted,^28^ comprising two anterior obliques beams(gantry 45 and 315), two lateral beams(gantry at 90 and 270 with 10‐degree deviation and possible couch rotation of 10 to 15 degrees) beams with a 4 cm range shifter, and two posterior obliques beams(gantry at 160 and 200). In addition, various beam‐specific blocks, including sinus and amalgam dental filling blocks for anterior beams, and shoulder blocks for posterior beams, were used to mitigate the range uncertainty due to anatomical variations. More details are described in ref. [28].

#### 2.2.1 Plan evaluation using accumulated dose metrics

We first performed rigid registration to align the vCT images with the planning CT, simulating the setup process for this cohort of patients. Then we simulated the effects of 3% range uncertainty and 1mm residual position uncertainty for each vCT. This residual uncertainty includes the coincidence between the treatment and imaging isocenters, the accuracy of the robotic table correction and the potential differences in rigid registration performed on the vCT in the TPS compared to the planning CT at the treatment gantry.^28^ This process generates 28 dose distributions for each vCT, consisting of a combination of 14 position and 2 range errors (please see Appendix B for illustration). Then, we calculated the voxmin and voxmax from the total 28 scenarios. Anaconda deformable image registration of RayStation was used to accumulate the voxmin, voxmax and nominal dose in the planning frame as the accumulated voxmin, accumulated voxmax and accumulated nominal dose. For high‐risk CTV, and low‐risk CTV, the metric used is the ratio of voxmin of V94 (V94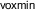), which is calculated by dividing the accumulated V94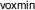 by the planning V94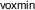 (ratio = accumulated V94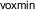/planning V94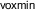). This metric allows for a fair comparison between strategies by accounting for slight differences in the initial CTV dose metrics at the planning stage. For brainstem, spinal cord, optical nerve and chiasm, the evaluation metric is the D0.03cc of the accumulated voxmax dose (${D0.03\mathrm{cc}}_{\text{voxmax}}$). For parotids (ipsilateral parotid and contralateral parotid), submandibular glands, pharyngeal constrictor muscles (split into superior, medius and inferior components), and oral cavity, the evaluation metric is the mean dose (Dmean) of the accumulated nominal dose.

To assess the statistical significance of OAR dose metric differences between each pair of strategies, we utilized the Wilcoxon test, a nonparametric method designed for comparing two related samples or paired groups.

The accumulated Dmean of bilateral submandibular and parotid glands was converted to NTCP for grade ≥ 2 patient‐rated xerostomia, and the accumulated Dmean of oral cavity and pharyngeal constrictor muscle (PCM) superior, medius, and inferior was converted to NTCP for grade ≥ 2 physician‐rated dysphagia according to the validated models of the NIPP for H&N cancer.^29^

(1) $$NTCP={\left(1+\exp\left(-S\right)\right)}^{-1}$$

$S_{\text{xerostomia}}$ for xerostomia and sticky saliva is given by S(patient‐rated xerostomia) = −2.2951 + 0.0182*(mean dose submandibular) + 0.0996*(√ mean dose ipsilateral parotid + √ mean dose contralateral parotid).

$S_{\text{dysphagia}}$ for dysphagia is given by S(patient‐rated dysphagia) = −4.0536 + 0.03*(mean dose oral cavity) + 0.0236*(mean dose PCM superior) + 0.0095 * (mean dose PCM medius) + 0.0133 * (mean dose PCM inferior) − 0.6281 * (if tumor location is pharynx).

## 3 RESULTS

**FIGURE 1:**
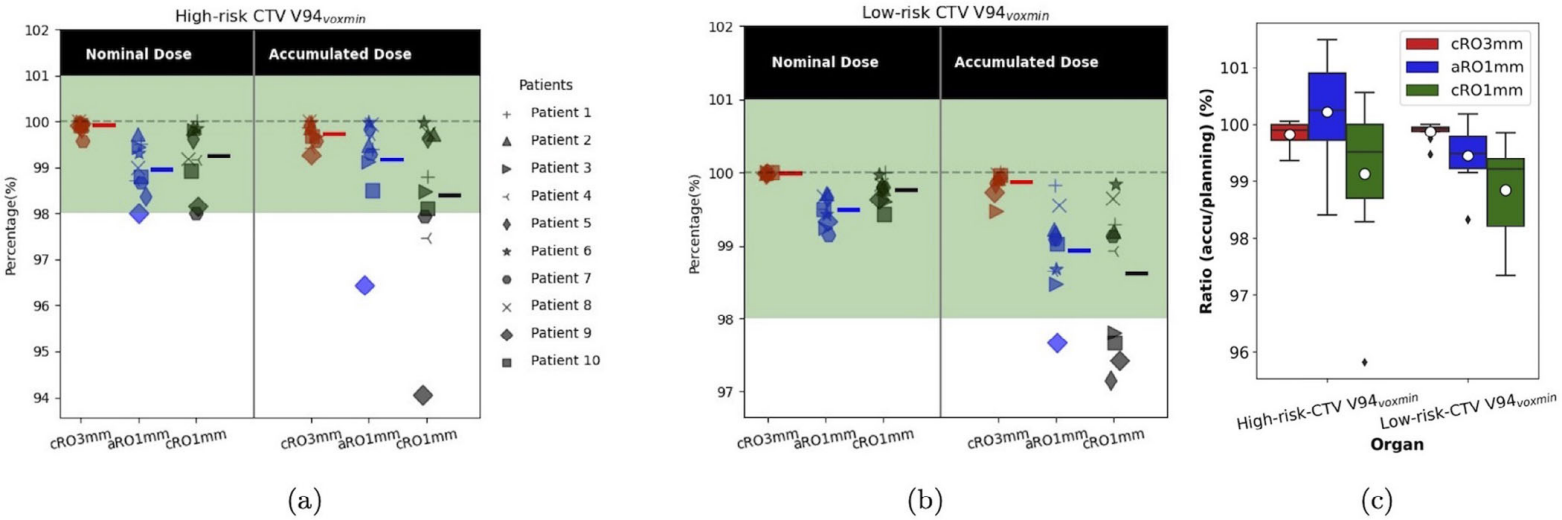
The comparison of three different strategies on CTV coverage based on the results of 10 patients. (a) and (b) shows the nominal planning dose and voxmin dose of accumulated dose on high‐risk CTV and low‐risk CTV for each individual, respectively. The green regions indicate the clinical goals. The dashed line on the right side of the scatter values represents their mean value. CTV, clinical target volume.

Figure 1a,b present the individual V94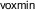 of the accumulated doses and the planning doses across three different strategies. Figure 1c statistically shows the ratio of accumulated V94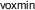 to planning V94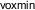 for CTVs in these strategies. Figure 1a shows the results of high‐risk CTV, the accumulated V94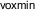 of cRO3mm are all above 98%, while aRO1mm has one case whose accumulated V94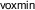 is 96.44% because its planning V94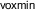 is right at 98%. The cRO1mm approach resulted in two cases with accumulated V94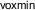 below 98%. The lowest accumulated V94voxmin observed was 94.05%, which approaches the threshold for triggering a replan. The mean values indicated by the dash on the right side of the scatter values show that, on average, the accumulated V94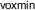 degraded slightly compared to the planning dose in both cRO3mm and cRO1mm, whereas in aRO1mm, the mean accumulated V94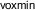 slightly exceeds the planned value. Figure 1b shows the results of low‐risk CTV. In cRO1mm, 4/10 patients experienced a decrease in V94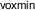 below 98% of total low‐risk CTV volume, with the lowest V94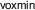 recorded at 97.15%. In contrast, aRO1mm has one case with a V94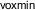 of 97.68%, and V94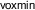 of every case in cRO3mm is above 98%. The ratio of the accumulated V94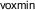 to the planning V94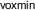 in Figure 1c reveals that, on average, aRO1mm achieves the highest ratio of V94voxmin for high‐risk CTVs, and maintains an average ratio above 99% for low‐risk CTVs.

**FIGURE 2:**
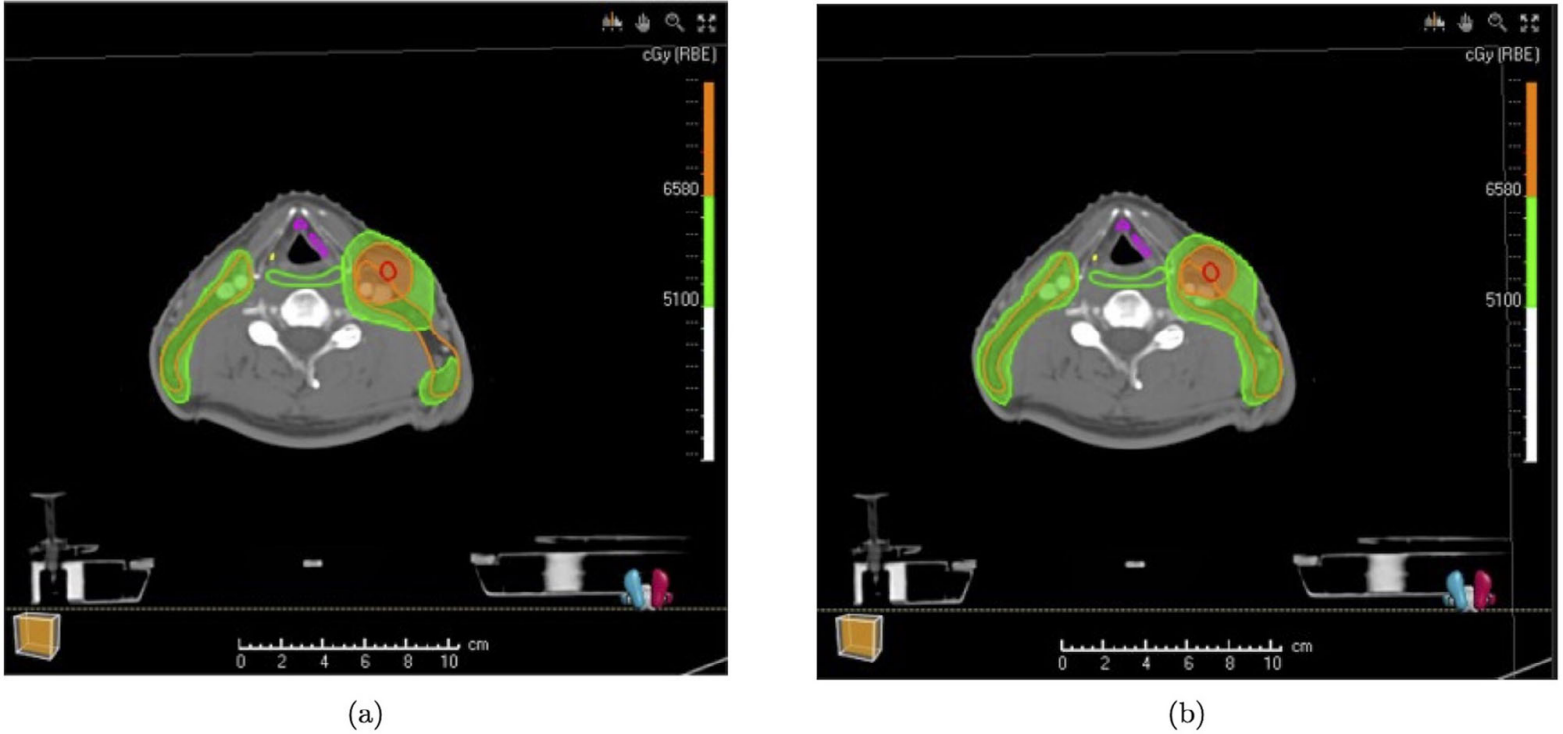
The comparison between cRO1mm and aRO1mm on the voxmin CTV coverage. (a) and (b) shows the accumulated voxmin dose distribution from cRO1mm and aRO1mm, respectively. The high‐risk CTV is delineated by red color. The low‐risk CTV is delineated by orange colour. The areas of the high‐risk CTV and the low‐risk CTV at and above 94% of prescription dose are highlighted in orange and green, respectively. CTV, clinical target volume.

In Figure 2, we show the accumulated voxmin dose for patient 5 to present CTV coverage by both aRO1mm and cRO1mm strategies. Because predicted images provide the additional changes in the neck area, low‐risk CTV is still covered well in the aRO1mm, even with 1mm setup uncertainty. Conversely, the cRO1mm strategy fails to deliver at least 94% of the prescribed dose to a specific area within the low‐risk CTV.

**FIGURE 3:**
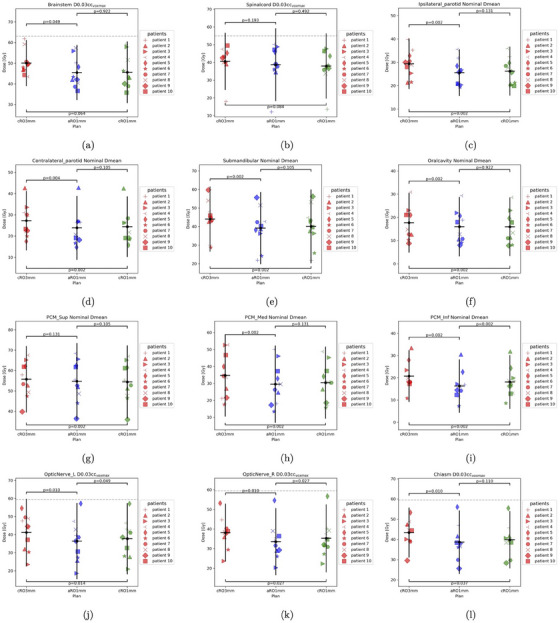
The comparison of three different strategies on OARs for 10 patients. For serial organs in (a)–(e), the dashed lines indicate the clinical goal. The horizontal line in the scatter data represents the mean value of the distribution, while the vertical line spans over its 95% confidential interval. The *p*‐values of the Wilcoxon test are calculated between strategies. OARs, organs at risk.

In Figure 3, we show the OAR dose metrics of individual cases for three strategies. The Wilcoxon test which compares two paired groups is used to calculate *p*‐values between any two of the strategies.

For all serial organs, $D_{\text{0.03cc}}$ values among patients are below the clinical constraints for three plan strategies. On average, $D_{\text{0.03cc}}$ values are lower in aRO1mm, cRO1mm, compared with cRO3mm.

For parallel organs, the average Dmeans in aRO1mm and cRO1mm is smaller than in cRO3mm. The reduction in Dmean of parallel organs when using aRO1mm compared to cRO3mm can reach 3.75 Gy, 3.37 Gy, 4.85 Gy, 1.70 Gy, 1.00 Gy, 5.22 Gy, 4.48 Gy for ipsilateral parotid, contralateral parotid, submandibular gland, oral cavity, PCM superior, PCM medius, and PCM inferior respectively. The Wilcoxon test results, comparing aRO1mm with cRO3mm, reveal *p*‐values less than the conventional significance level of 0.05 for all parallel organs except for PCM superior, highlighting the benefits of aRO1mm in terms of sparing OARs. The *p*‐values between aRO1mm and cRO1mm are all above 0.05, except for PCM inferior (0.002), showing that the distributions of Dmean from aRO1mm and cRO1mm are mostly similar.

The original data of the planning and accumulated dose for each patient are listed in the supplementary table of dataset.xlsx.

**FIGURE 4:**
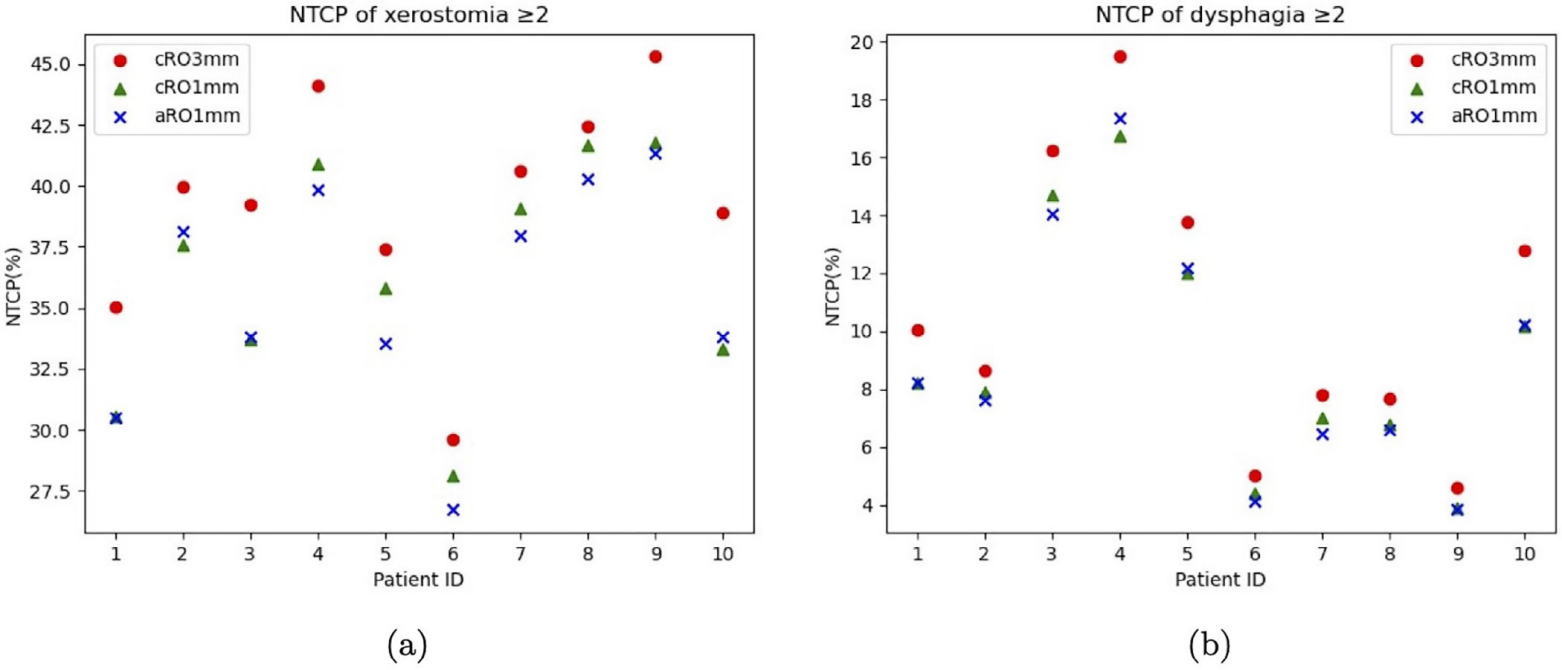
The comparison between the NTCPs of cRO3mm and aRO1mm on for the ten validation patients. (a) The NTCP of grade ≥ 2 patient‐rated xerostomia. (b) The NTCP of grade ≥ 2 patient‐rated dysphagia. NTCP, normal tissue complication probability.

We evaluated the NTCP of grade ≥ 2 patient‐rated xerostomia and grade ≥ 2 patient‐rated dysphagia for each patient in Figure 4. Compared with cRO3mm, the maximum reduction in the NTCP of grade ≥ 2 xerostomia by using the aRO1mm strategy is 5.41% with an average reduction of 3.67%. Similarly, the maximum reduction in the NTCP of grade ≥ 2 xerostomia by using the aRO1mm strategy is 2.57%, with an average reduction of 1.54%.

## 4 DISCUSSION

The novelty of this research lies in its pioneering incorporation of a predictive anatomy model directly in IMPT robust optimization for nasopharyngeal cancer patients. By this approach, the possibility of reducing setup uncertainty to actual simulated setup uncertainty of 1mm was explored to reduce the doses and ultimately the complication probability of the normal tissues.

In this study, we used a setup uncertainty of 1mm in the robust optimization setting. However, the concept presented here can be adapted to different set‐up settings used in the treatment centers because in this study we simulated the setup error based on our assumption of 1mm position uncertainty. Our key finding reveals that robust optimization considering only setup uncertainty (as in cRO1mm) fails to adequately address tumor coverage degradation caused by anatomical changes. While increasing the setup uncertainty beyond the true 1mm value (as in cRO3mm) can improve dose coverage in the presence of anatomical changes, this approach results in undesirable overdosage to OARs, as illustrated in Figure 4. We introduce a novel method that uses additional images to represent anatomical uncertainty. By incorporating these images into robust optimization, along with the actual 1mm setup uncertainty, we achieve improved tumor coverage while better sparing OARs. This approach demonstrates the benefits of accurate uncertainty modeling, as it accounts for both setup and anatomical uncertainties without compromising OAR protection.

The impact of using the aRO1mm strategy on the NTCP for OARs is notably contingent upon the specific NTCP model employed. Previous studies^30^ reported that 3 Gy for parotids (both ipsi‐ and contralateral parotid glands included) would result in NTCP differences of 3%–10% for xerostomia (depending on the applied model and the steepness of the curve for the particular dose value). Such a reduction is considered clinically significant, serving as a threshold for replanning in TORPEdO trial (a phase III trial of proton therapy versus intensity‐modulated radiotherapy for multitoxicity reduction in oropharyngeal cancer; CRUK/18/010).^31^ In our analysis, the reduced parotid dose with aRO1mm reached this threshold.

Compared with the mean reduced NTCP of grade ≥ 2 xerostomia of 3.67% from using the aRO1mm strategy, the mean reduced NTCP of grade ≥ 2 dysphagia is only 1.54% primarily because of the limited dose reduction in the PCM superior from aRO1mm. In most studied patients, the PCM superior largely overlapped with the high‐risk CTV, making it difficult to spare the small nonoverlapped portion despite the maximal freedom offered by the six beam arrangement without beam‐specific block at this target level.

Both aRO1mm and cRO1mm have the worst CTV target coverage degradation on patient 9. The ratios of high‐risk CTV V94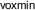 are 98.41% and 95.82% in aRO1mm and cRO1mm, respectively. The ratios of low‐risk CTV V94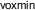 are 98.33% and 97.79% in aRO1mm and cRO1mm, respectively. The aRO1mm had lower target coverage degradation compared to cRO1mm. The deviation was attributed to a consistent setup error related to the patient's shoulder position presented on the vCT. Specifically, in the planning CT scan, the patient's left shoulder was positioned higher than the right. On all vCTs, the left shoulder was consistently aligned with the right shoulder horizontally, differing from the planning position (see supplementary materials in Appendix C). Although immobilization masks can assist in patient positioning, minor downward movement of the shoulders remains possible. In the absence of cone‐beam CT (CBCT), it is challenging to be sure if vCTs can represent the patient's actual position during the treatment. Nevertheless, based on the observed consistency across multiple vCTs and probabilistic reasoning, we can reasonably infer that the vCT images more closely approximate the patient's true position during treatment delivery. Because the average model primarily accounted for the systematic progressive changes, random position changes that were left out in the predicted images could still lead to dose degradation on CTV. Although we aligned the vCT to the planning CT following the IGRT rigid registration protocol as closely as possible for dose calculation, the influence of small nonrigid positioning errors cannot be fully removed and hence the results are considered reasonable under these circumstances.

In clinical situations where small neck tilt and shoulder movement cannot be completely eliminated with IGRT, leading to dose degradation as encountered in patient 9, a potential solution is to incorporate additional predicted images that capture these random variations. Zhang et al.^13^ employed principal component analysis (PCA) to generate weekly predicted images of NPC patients, specifically to account for random positioning changes. Their study demonstrated that the PCA‐based probability model more accurately represents anatomical uncertainty compared to the AM. Other deep‐learning‐based probabilistic models are also expected to augment the prediction of anatomical changes and thereby further improve the plan robustness.^32^

This study uniformly included three additional predicted anatomies at the 1st, 3rd, and 5th week in aRO1mm, which was in line with findings from a recent study suggesting the third week as the optimal timing for replanning and a maximum of three replannings can guarantee the increase of mean dose below 3 Gy for swallowing and salivary organs in photon‐based radiotherapy.^33^ Adding more predicted images may not be clinically practical as it may significantly increase the optimization time despite the availability of a GPU‐accelerated Monte Carlo dose engine. For the current aRO1mm approach using 3 additional images, there are 112 scenarios to be optimized (14 set error scenarios + 2 range error scenarios per image set). Also, the plan robustness with more predicted images might come at the cost of plan dosimetry.^22^ Future work should explore the balance between increased robustness from using more predicted images and overall plan quality. This would help determine the optimal number of images to include in the optimization process.

In the cRO1mm strategy, 6/10 cases can meet the clinical goals for CTV coverage with 1mm setup uncertainty used in robust optimization. These patients have relatively small progressive changes. If an individual's changes can be predicted accurately before treatment by identifying features that might be related to the anatomical changes,^34^, ^35^, ^36^, ^37^, ^38^ we can incorporate only predicted images for patients with larger anatomical changes to limit the computational overhead.

Currently, the predictive ability of the model is constrained by a relatively small dataset of 20. In this circumstance, a slight increase of the actual setup uncertainty, for example, to 1.5mm, might help with the target coverage at the tradeoff of a slight increase in the NTCP. The key takeaway is that the magnitude of reduction in rigid shift simulation in robust optimization can be finely tuned according to the accuracy of a predictive model. The more precise the model, the more confidently we can use the actual position uncertainty observed at the treatment center.

In the clinic, the magnitude of uncertainty is typically derived from data across a patient population.^14^, ^39^, ^40^, ^41^ Scandurra et al.^28^ reported that plan adaptation due to loss of target coverage is low (2/25) using the cRO3mm strategy as in this study. Therefore, our patient cohort represented the most patient cases and our conclusions apply to the majority of clinical scenarios. Despite the limited number of studied patients, the validation of our proof‐of‐concept of aRO1mm, by comparing with cRO3mm, clearly supports that the systematic interfractional changes as presented by the predicted images of AM are more influential than conformal shifts in terms of the sparing dose of OARs.

In this study, none of our patients required replanning based on our protocol (V94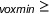 94%), even though we already included the patients with the largest anatomical changes of all our patients (*n* = 20) used to build AM. However, our method has the potential to be used for replanning patients. Cubillos‐Mesias et al^42^ showed that plan adaptation can be reduced with additional images, presenting the benefits of including anatomical uncertainty in the robust optimization. However, there may be a trade‐off between set‐up error reduction and the number of plan adaptations, which warrants further investigation.

We used 10 patients with weekly CT imaging, which is used less frequently in routine clinics than cone‐beam CT (CBCT), to reduce the error of converting CBCT to synthetic CT for dose evaluation.^43^, ^44^ The procedure of using CBCT images to build the model is the same except that the influence of DIR between CT and CBCT might be different.^45^

## 5 CONCLUSION

This study underscores the potential of predictive imaging to enhance radiation therapy planning by accurately accounting for patient‐specific anatomical variability throughout treatment, thereby optimizing the balance between effectively targeting the tumor and minimizing exposure to surrounding healthy tissues. Different strategies for using predictive models will be explored for various H&N cohorts, such as patients who require replanning, in the future.

## CONFLICT OF INTEREST STATEMENT

The authors declare no conflicts of interest.

## Supporting information

Supporting Information

Supporting Information

## Data Availability

The authors cannot make these data publicly available due to data use agreement. The authors only accessed the imaging data whose identifiers were removed. This study nor need interaction with the patients or involves clinical intervention. Therefore IRB approvals per study institutions are not required.
