# Supplementary material for: Clinical advantages of incorporating predicted weekly anatomy in IMPT optimization with reduced setup error
Source: Med Phys. 2024 Sep 19;51(12):9207–16. doi: 10.1002/mp.17412 (PMC11656292; doi:10.1002/mp.17412)
Supplement: Supplementary file 1 — Supporting Information [file MP-51-9207-s001.pdf]

Appendix A: Robust optimisation details

The details of robust optimisation algorithm can be found in the official document of Raystation. Basically, anatomical robust optimisation (aRO) adapts the same idea as conventional robust optimisation using setup error and range error. In the aRO, the uncertainty is extended to the additional images. In our case, using 1 planning CT + 3 predicted images, the number of error scenarios increases to 4 (number of image sets) X 14 setup error scenarios x 2 range error scenario. To be noted, the contours on the predicted CT would be used in the robust function. The minimax robust optimization algorithm will search for a solution where the resulting robust objectives can be achieved as far as possible in all these scenarios (different anatomical CTs, setup error and range error), such as brainstem dose constraints need to be satisfied on the predicted CT using its predicted contours.

Appendix B: The illustration of 28 scenarios for dose distribution calculation

The 28 scenarios for calculating the perturbed dose distributions under a setup and a range uncertainty are shown in figure 2.

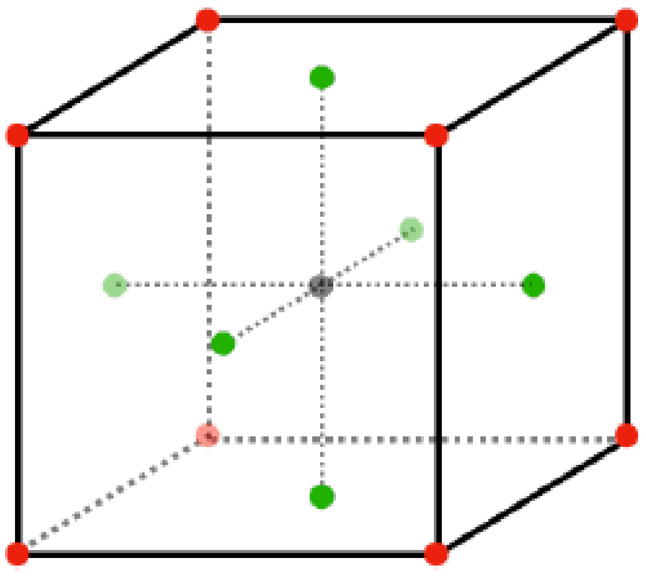

FIG. 1: The setup error in directions. 6 principal directions (6 green dots) and from the centre to the vertices(8 red dots), the combinations of these including ±3.5% range uncertainty result in 28 scenarios.

Appendix C: The illustration of setup error for patient 8

The setup error of patient 8. The figure shows the image difference between planning CT and rigid registered weekly CT. In the shoulder area. The red area of the left shoulder and the blue area of the right shoulder demonstrate that in the planning, the left shoulder is higher than the right shoulder.

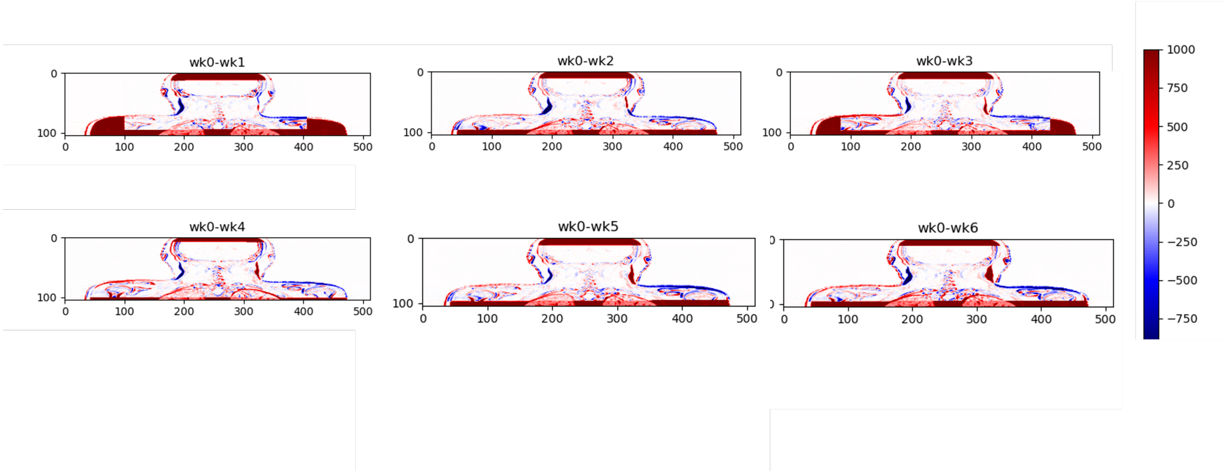

FIG. 2: The illustration of setup error for patient 8
